# Crohn’s Disease Presenting as an Abdominal Wall Abscess in an Adolescent With Autism Spectrum Disorder

**DOI:** 10.1155/crpe/8225541

**Published:** 2026-09-22

**Authors:** Kohsuke Hitomi, Shuichi Katayama, Soichi Nakada, Shojiro Hanaki

**Affiliations:** ^1^ Department of General Surgery, Division of Pediatric Surgery, Kurashiki Central Hospital, Kurashiki, Japan, kchnet.or.jp

## Abstract

Crohn’s disease (CD) is a subtype of inflammatory bowel disease characterized by discontinuous lesions and transmural inflammation. Delayed diagnosis may lead to fistula formation between the intestine and adjacent organs. However, most CD‐associated fistulas are enteroenteric or perianal, and abdominal wall abscesses are rarely reported in pediatric patients. We report a case of CD presenting as an abdominal wall abscess in a 15‐year‐old girl with autism spectrum disorder. Contrast‐enhanced computed tomography revealed a fistulous communication between the abscess cavity and the transverse colon. Percutaneous drainage and antibiotic therapy were performed to control the infection. Colonoscopy demonstrated skip lesions with longitudinal ulcers, cobblestone‐like mucosa, and inflammatory polyps. Histopathological examination showed active chronic colitis compatible with inflammatory bowel disease. Remission induction therapy consisting of elemental diet, 5‐aminosalicylic acid, and corticosteroids was initiated after infection control. Her clinical condition gradually improved, and she was discharged on hospital Day 37. During follow‐up, her appetite improved and she gradually gained weight. At 18 months after discharge, contrast‐enhanced computed tomography showed no recurrence of the abdominal wall fistula, and her clinical condition remained stable. In pediatric patients, CD may present with nonspecific systemic symptoms, which can delay diagnosis. In patients with autism spectrum disorder, communication difficulties may further obscure gastrointestinal symptoms. Clinicians should consider underlying inflammatory bowel disease when children present with unexplained abdominal wall abscesses accompanied by systemic symptoms such as growth failure or malnutrition.

## 1. Introduction

Crohn’s disease (CD) is a chronic inflammatory disorder that can affect any part of the gastrointestinal tract and is commonly diagnosed based on symptoms such as abdominal pain, chronic diarrhea, hematochezia, and weight loss. Because CD causes transmural inflammation, delayed diagnosis and disease progression may lead to fistula formation between the intestine and adjacent organs [1].

Although fistula formation is a well‐recognized complication of CD, most fistulas are enteroenteric or perianal. Abdominal wall abscesses caused by fistulizing CD are rare, particularly in pediatric patients.

Moreover, several factors may contribute to delayed diagnosis of CD in patients with autism spectrum disorder, including communication difficulties and atypical symptom expression [2].

In this report, we present a rare case of CD diagnosed following the development of an abdominal wall abscess in an adolescent with autism spectrum disorder.

## 2. Case Presentation

The patient was a 15‐year‐old girl who had been diagnosed with autism spectrum disorder in the third grade of elementary school. At age 13 years, she was admitted to the pediatric department of our hospital because of poor oral intake, weight loss, and malnutrition and required 2 months of inpatient nutritional management. One week before presentation, she developed decreased appetite, followed 3 days later by fever. She then noticed a right‐sided abdominal mass and presented to our hospital.

On admission, she was small and underweight, with a height of 145.0 cm (−2.0 SD), weight of 30.8 kg (−2.6 SD), and body mass index of 14.6 kg/m^2^. Her body temperature was 39.5°C. Abdominal examination revealed a soft but slightly tender mass approximately 6 cm in diameter in the right upper abdomen without overlying skin erythema.

**FIGURE 1:**
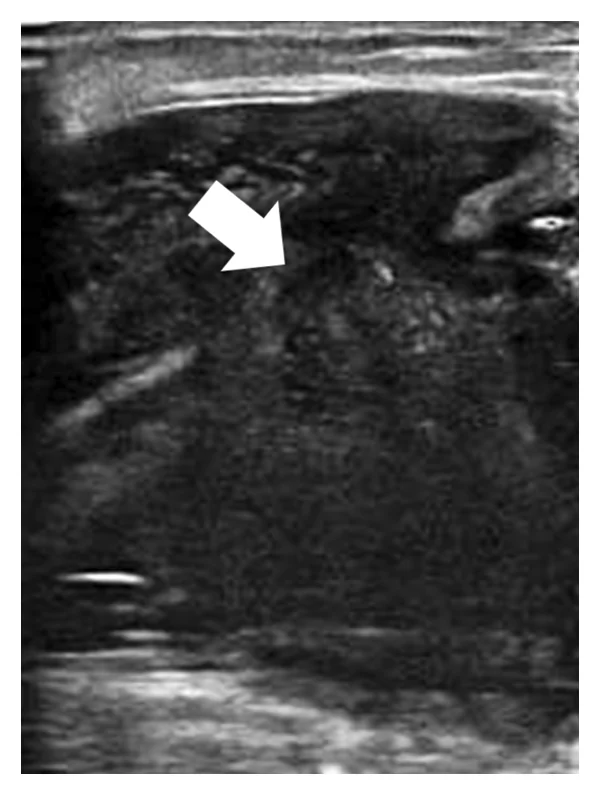
Abdominal ultrasonography demonstrating an abdominal wall abscess with a fistulous tract extending through the abdominal wall musculature and communicating with the peritoneal cavity (arrow).

**FIGURE 2:**
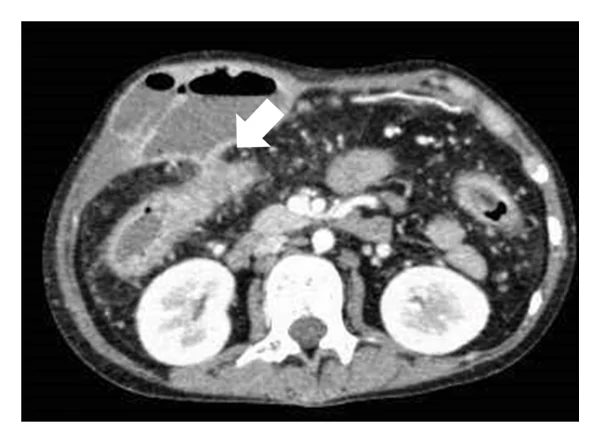
Contrast‐enhanced computed tomography demonstrating a fistulous communication between the abscess cavity and the transverse colon (arrow), with circumferential thickening of the colonic wall.

Abdominal ultrasonography revealed a 7.8 × 6.1‐cm subcutaneous abscess with a 1.6‐cm fistulous tract extending through the abdominal wall musculature and communicating with the peritoneal cavity (Figure 1). Contrast‐enhanced computed tomography demonstrated a fistulous communication between the abscess cavity and the transverse colon, along with circumferential wall thickening of the colon (Figure 2). In addition, small intra‐abdominal abscesses measuring 1.5 cm and 2.0 cm were observed near the splenic flexure and the dorsal aspect of the sigmoid colon. Laboratory tests showed leukocytosis, elevated inflammatory markers, anemia, and marked hypoalbuminemia (Table 1).

**TABLE 1:** Laboratory findings on admission.

		Normal range
Leukocytes	12.7 × 10^3^/μL	3.3–8.6 × 10^3^/μL
Hemoglobin	9.9 g/dL	11.6–14.8 g/dL
Thrombocytes	67.4 × 10^4^/μL	16.0–36.0 × 10^4^/μL
CRP	6.86 mg/dL	0.00–0.14 mg/dL
TP	6.4 g/dL	6.6–8.1 g/dL
Alb	1.6 g/dL	4.1–5.1 g/dL
ChE	62 U/L	201–421 U/L
AST	18 U/L	13–30 U/L
ALT	10 U/L	7–23 U/L
Creatinine	0.31 mg/dL	0.46–0.79 mg/dL
UN	8 mg/dL	8–20 mg/dL

*Note:* Alb, albumin; ChE, cholinesterase; AST, aspartate aminotransferase; ALT, alanine aminotransferase.

Abbreviations: CRP, C‐reactive protein; TP, total protein; UN, urea nitrogen.

**FIGURE 3:**
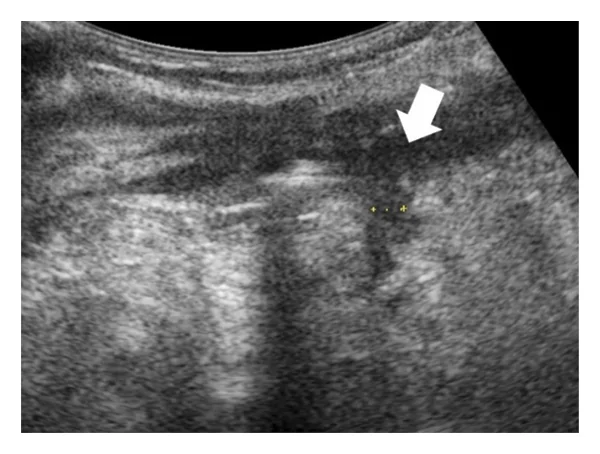
Follow‐up abdominal ultrasonography after initial treatment demonstrating shrinkage of the abdominal wall abscess and marked reduction of the fistulous tract (arrow).

Based on these findings, an abdominal wall abscess was diagnosed, and inflammatory bowel disease, particularly CD, was suspected as the underlying condition. Initial management consisted of percutaneous drainage of the abdominal wall abscess and administration of broad‐spectrum antibiotics (tazobactam/piperacillin). The patient subsequently became afebrile, and inflammatory markers gradually improved. Follow‐up ultrasonography demonstrated shrinkage of both the abscess cavity and the fistula (Figure 3), and oral intake was resumed on hospital Day 6.

**FIGURE 4:**
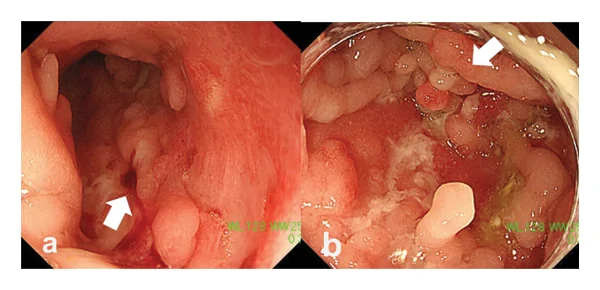
Colonoscopic findings. (a) Longitudinal ulcers with edematous mucosa forming a cobblestone‐like appearance (arrow). (b) Multiple inflammatory polyps consistent with active Crohn’s disease (arrow).

Colonoscopy was performed on hospital Day 8 and revealed discontinuous inflammatory lesions from the sigmoid colon to the descending colon. The affected mucosa showed erythema with longitudinal ulcers, inflammatory polyps, and a cobblestone‐like appearance (Figure 4), findings suggestive of CD.

Histopathological examination of colonic biopsy specimens revealed crypt architectural distortion, cryptitis, and crypt abscess formation. Moderate inflammatory cell infiltration was observed in the lamina propria, predominantly consisting of lymphocytes and plasma cells with admixed eosinophils and neutrophils. Immunohistochemical staining for cytomegalovirus was negative. These findings were consistent with active chronic colitis compatible with inflammatory bowel disease. Based on the clinical presentation, endoscopic findings, and histopathological results, a diagnosis of CD was made.

Enteral nutrition with elemental diet (Elental) and 5‐aminosalicylic acid (5‐ASA) were initiated on hospital Day 10. The drainage catheter was removed and antibiotic therapy was discontinued on hospital Day 15 after confirmation of infection control. On hospital Day 18, oral prednisolone (PSL) was started for remission induction, and an inflammatory bowel disease diet was initiated.

After treatment initiation, the patient’s clinical condition gradually improved. No recurrence of the abscess or fistula was observed, and oral intake remained stable. The patient was discharged on hospital Day 37.

After discharge, she continued to be followed regularly by the gastroenterology department. Although complete remission had not yet been achieved, her appetite improved and she gradually gained weight. At 18 months after discharge, contrast‐enhanced computed tomography demonstrated no recurrence of the abdominal wall fistula or intra‐abdominal abscess, and her clinical condition remained stable.

## 3. Discussion

CD is a subtype of inflammatory bowel disease that primarily affects the terminal ileum and colon although it can involve any part of the gastrointestinal tract. It is characterized by discontinuous (skip) lesions and transmural inflammation. When inflammation penetrates deeply into the intestinal wall, fistulas may form between intestinal segments or between the intestine and adjacent organs, including the skin [1].

CD is typically diagnosed based on persistent gastrointestinal symptoms such as abdominal pain, chronic diarrhea, and hematochezia; systemic manifestations including fever, fatigue, and anemia; and abnormal laboratory findings such as elevated inflammatory markers and hypoalbuminemia. Perianal disease, including fistulas or strictures, may also be present [3]. In pediatric patients, however, gastrointestinal symptoms may be less prominent, and growth failure or delayed development may be the initial manifestation. Compared with adults, nonspecific systemic signs are often more apparent, which may contribute to delayed diagnosis [4]. In addition, the chronic and relapsing nature of the disease may lead to transient symptom improvement, further delaying definitive diagnosis [5].

Abdominal wall abscess as the initial manifestation of CD is extremely rare, particularly in pediatric patients. In the present case, the diagnosis of CD was prompted by an abdominal wall abscess, which likely resulted from fistula formation secondary to transmural intestinal inflammation. The patient had previously been hospitalized at age 13 years for weight loss, malnutrition, and growth failure, suggesting that CD may already have been present but remained undiagnosed. Several factors may have contributed to the delayed diagnosis in this patient. These included limited awareness that pediatric CD may present with nonspecific symptoms and difficulties in clinical assessment and diagnostic evaluation related to the patient’s coexisting autism spectrum disorder. Furthermore, decreased food intake may have been mistakenly attributed to selective eating behaviors or food preferences.

The patient had been diagnosed with autism spectrum disorder prior to the diagnosis of CD. Previous studies have suggested that the prevalence of inflammatory bowel disease is higher in individuals with autism spectrum disorder than in the general population [6]. The mechanisms underlying this association are thought to involve a complex interaction of factors, including immune dysregulation, altered intestinal microbiota, disruption of the gut–brain axis, overlapping genetic predispositions, and impaired intestinal environments related to selective eating behaviors [2]. In addition, communication difficulties and atypical expressions of pain or discomfort may obscure gastrointestinal symptoms, increasing the risk of delayed diagnosis. Therefore, clinicians should maintain a high index of suspicion for inflammatory bowel disease in children with autism spectrum disorder who present with systemic symptoms such as weight loss, malnutrition, or growth failure.

The reported incidence of intra‐abdominal abscesses in CD ranges from approximately 10%–30% [7]. Abscess formation represents a consequence of transmural inflammation and fistula development rather than a simple infectious complication [8]. Fistulizing CD is recognized as a marker of disease progression and is associated with a high risk of recurrence and the need for surgical intervention. Risk factors include early onset of disease, perianal involvement, and small bowel disease [7]. In the long term, approximately 30%–50% of the patients with CD require surgical procedures such as bowel resection, stricturoplasty, or stoma creation [9]. Pediatric‐onset CD is often associated with a more aggressive clinical course than adult‐onset disease, with an increased risk of fistula formation and intestinal strictures. The deterioration of general health due to growth failure further underscores the importance of early diagnosis and timely treatment in children [10].

In the present case, infection was successfully controlled through percutaneous drainage and broad‐spectrum antibiotic therapy without surgical intervention. After stabilization of the patient’s systemic condition, medical treatment for CD was initiated, leading to spontaneous closure of the fistula. The clinical course suggests that treatment strategies similar to those used for intra‐abdominal abscesses may also be effective in managing abdominal wall abscesses associated with CD. During 18 months of follow‐up, no recurrence of the fistula or abscess was observed, and the patient maintained a stable clinical condition with outpatient medical management. Nevertheless, because fistulizing CD is associated with a risk of recurrence and disease reactivation [11], long‐term multidisciplinary follow‐up remains essential.

This case provides several important clinical insights. First, CD may initially present with an abdominal wall abscess although this presentation is extremely rare in pediatric patients. Second, the coexistence of autism spectrum disorder may obscure gastrointestinal symptoms and contribute to delayed diagnosis. Third, effective infection control using percutaneous drainage and antibiotics may allow safe initiation of medical therapy without immediate surgical intervention. When pediatric patients present with unexplained abdominal wall abscesses accompanied by systemic symptoms such as weight loss, malnutrition, or growth failure, clinicians should consider the possibility of underlying inflammatory bowel disease. Early recognition and appropriate multidisciplinary management may help prevent disease progression and serious complications.

## Funding

No funding was received for this manuscript.

## Ethics Statement

Ethical approval was waived by our institutional review board because this study describes a single case report.

## Consent

Written informed consent was obtained from the patient’s guardian for publication of this case report and the accompanying images.

## Conflicts of Interest

The authors declare no conflicts of interest.

## Data Availability

The data that support the findings of this study are available from the corresponding author upon reasonable request.
